# Single fraction of accelerated partial breast irradiation in the elderly: early clinical outcome

**DOI:** 10.1186/s13014-018-1119-6

**Published:** 2018-09-12

**Authors:** Rémy Kinj, Marie-Eve Chand, Jocelyn Gal, Mathieu Gautier, Lucile Montagné, Daniel Lam Cham Kee, Jean Michel Hannoun-Lévi

**Affiliations:** 1 Department of Radiation Oncology, Centre Antoine Lacassagne - University of Cote d’Azur, 33 avenue de Valombrose, 06000 Nice, France; 2 Biostatistic unit, Centre Antoine-Lacassagne, 33 av de Valombrose, 06189 Nice, France

**Keywords:** Breast cancer, Elderly, Accelerated partial breast irradiation, Brachytherapy

## Abstract

**Background:**

To analyze the clinical outcome of elderly women with early breast cancer who underwent accelerated partial breast irradiation (APBI) based on a post-operative single fraction of multicatheter interstitial high dose–rate brachytherapy (MIB).

**Methods:**

A single institution retrospective cohort study was performed focusing on elderly patients (≥ 65 years old) presenting a low-risk breast carcinoma treated by lumpectomy plus axillary evaluation followed by MIB. A single fraction of 16 Gy was prescribed on the 100% isodose. Clinical outcome at 3 years was reported based on local relapse free survival (3-y LRFS), specific survival (SS) and overall survival (OS). Acute (< 180 days after APBI) and late toxicity were evaluated. Cosmetic results were clinically evaluated by the physician.

**Results:**

Between January 2012 and August 2015, 48 women (51 lesions) were treated. Median age was 77.7 years (range: 65–92) with a median tumor size of 12 mm (range: 3–32). Five patients (pts) presented an axillary lymph node involvement (4 Nmic, 1 N1). Invasive ductal carcinoma was the most frequent histology type (86.3%). With a median follow–up of 40 months (range: 36–42), no local relapse occurred while 1 pt. developed axillary relapse (2.1%). The 3-y LRFS, SS and OS rates were 100%, 100% and 93.1% respectively. Forty-five acute events were remained. The most frequent acute toxicity was grade (G) 1 hyperpigmentation (26.7%), 3 pts. (6.3%) presented G3 acute toxicity (2 breast hematomas, 1 breast abscess). No ≥ G3 late toxicity was observed while 15 late toxicities occurred (G1: 13 events - 86.7%) mainly breast fibrosis). The rate of excellent cosmetic outcome was 76.4%.

**Conclusion:**

We reported promising and encouraging clinical outcome of a post-operative single fraction of MIB ABPI in the elderly. This approach leads to consider a sfAPBI as an attractive alternative to intra-operative radiation therapy while all the patients will be good candidates for APBI in regards to the post-operative pathological report. More mature results (number of patients and follow-up) are needed.

## Background

During the last half of century, breast cancer therapeutics progressed steadily and rapidly. In the management of localized breast cancers, total mastectomy has gradually given way to conservative surgical treatments followed by adjuvant radiotherapy. The conservative therapeutic approach is now considered as a standard of care for T1–2 breast cancer [1, 2]. However, the standard adjuvant radiotherapy schedule (25 to 30 fractions) generates much transportation that can be difficult mainly for elderly women. Even using hypofractionated regimen (15 to 16 fractions) [3–5] in place of standard schedule, adjuvant breast irradiation can alter the quality of life and sometimes the adhesion to the treatment. Furthermore, a higher number of transportations generates additional costs for health insurance.

Accelerated partial breast irradiation (APBI) appears as a natural continuity in the process of therapeutic de-escalation. American Society of Radiation Oncology (ASTRO) and Groupe Européen de Curiethérapie of the European Society for Radiotherapy and Oncology (GEC-ESTRO) have considered that, in a well selected population, described as “suitable” (ASTRO) and “low-risk” (ESTRO), adjuvant APBI can be proposed [6, 7]. After two decades of clinical research, APBI is now recognized as an efficient and safe adjuvant treatment for low-risk breast cancer [8, 9].

The aim of this study was to report the early clinical outcome of APBI in the elderly with early breast cancer treated by a post-operative single fraction of MIB APBI (sfAPBI).

## Methods

### Patient selection

This is a single institution retrospective study including elderly patients presenting with low-risk breast cancer who underwent lumpectomy plus axillary evaluation followed by a single fraction of high-dose rate (HDR) MIB APBI. The patient cohort combined women enrolled in a prospective phase I/II trial (SiFEBI; Clinical.gov #NCT01727011, [10]) and patients previously treated before the SiFEBI trial opening. Briefly, inclusion criteria were as follows: elderly women 65 years and older, histologically proven breast carcinoma with free surgical margins, negative axillary evaluation. Patients were excluded in case of: sarcoma or lymphoma histology, metastatic dissemination. Data were collected from the Antoine Lacassagne Cancer Center institutional database. All the patients treated out of the SiFEBI trial, had the choice between adjuvant WBI and sfAPBI. All APBI indications were validated by the local breast oncologist committee. Patients treated outside of the SiFEBI trial (Clinical.gov #NCT01727011) were carefully selected and fully informed about the new irradiation procedure (advantages and disadvantages) compared to the standard protocol of external beam radiation therapy currently used in our institution. All those patients signed consent form before starting the treatment.

### Treatments

#### Breast surgery

As previously described*,* axillary dissection concerned Level I and II axillary lymph node area while sentinel lymph node biopsy alone was also achieved with per-operative exam and conversion to axillary dissection in case of positive biopsy. Then, lumpectomy was performed. Quality of margins was assessed by a per-operative pathological exam. Four to five clips were clamped by the surgeon to mark the tumor bed before closing the tumor bed cavity [11].

#### Brachytherapy

Brachytherapy was performed according to the GEC-ESTRO Breast Cancer Working Group recommendations for MIB APBI [12]. As previously described, vectors (Sharp Needles™; Elekta AB, Stockholm, Sweden) were placed mainly intra-operatively by the radiation oncologist using 1 to 3 planes in respect with Paris system recommendations [11]. Two days after the implant, a post implant CT (2.5 mm thickness slice) was performed in order to delineate the clinical target volume (CTV) based on clips and surgical cavity (if visible) including a total safety margin of about 2 cm (sum of the resection margin size and “added” safety margins size) [13]. Then, the dose distribution was optimized manually (OncentraBrachy®; Elekta, Sweden) by varying time and stop position of the radioactive source. A single fraction of 16 Gy was prescribed to the 100% isodose. This dose was calculated considering an α/β ratio of 3.4 Gy for breast late toxicity and 4.6 for local control [14]. According to the linear quadratic model, the equivalent dose at 2 Gy (EQD2) for a single fraction of 16 Gy is equal to 53 Gy with a α/β = 4 Gy [15, 16]. Dose constraints were as follow: D90% ≥ 105% of the prescribed dose, D100% ≥ 75%, V100 > 95% of the CTV, V150 ≤ 40%, V200 ≤ 15%; dose non-homogeneity ratio (DNR) ≤ 35% [10]. For organ at risk (skin and thoracic wall), the maximum skin-dose was < 75% of the prescribed dose while the maximum rib dose was < 100% of the prescribed dose.

#### Systemic therapy

Systemic therapies such as adjuvant chemotherapy and/or hormonal treatments were dispensed according to the protocols used in the Antoine Lacassagne Cancer Center.

### Follow up

The radiation oncologist performed iterative monthly post-brachytherapy clinics during 3 months (acute brachytherapy side effects). Then, clinical surveillance was performed alternatively with the surgeon twice a year with a yearly mammogram. Acute (< 180 days after treatment) and late toxicities were evaluated by Common Terminology Criteria for Adverse Event v3 (CTCAE.V3.0) [17]. Cosmetic evaluation was performed according to Harvard criteria [18].

### Statistical analysis

Description of the study population and of the different investigated parameters was made using absolute and relative frequencies for the qualitative data and summarized using descriptive statistics such as median, extreme for quantitative data. Survival time was defined between the surgery date and the event date. Local relapse free-survival (LRFS), regional relapse free-survival (RRFS), specific (SS) and overall survivals (OS) were estimated using the Kaplan-Meier method. Patients still alive were censored at the date of last follow-up. Median follow-up with 95% confidence intervals was calculated by reverse Kaplan–Meier method. Data entry and data management were performed on Ennov clinical® system and were analyzed using R 3.2.2 for Windows®.

## Results

### Patient and tumor characteristics

Between January 2012 and August 2015, a total of 51 lesions from 48 patients (pts) were treated with a sfAPBI. Among these patients, 26 were part of the SiFEBI trial while 22 pts. were treated out of the phase II study. Patient, tumor and treatment features are detailed in Table 1. Patient median age was 77.7 years [range: 65–92]. Most of patients were ECOG Performans Status (PS) 0 (85%). The most frequent location was the upper external quadrant (39.2%). Histological type was mainly invasive ductal carcinoma (86.2%). The median tumor size was 12 mm [range: 3–32] while, 4 pts. presented with a microscopic node involvement (Nmic) and 1 pt. was classified N1. The median surgical margin was 5 mm [range: 1–10]. One lesion was associated with peri-neural invasion. All the tumors but three had positive hormonal receptor status while Her-2 status was over-expressed in 9.8%.

**Table 1:** Patients, lesions and treatment characteristics

Patient features	Number of patients	% / (min – max)
Patients included in SiFEBI trial		
Yes	26	54.2
No	22	45.8
Mean age (years)	77.7	(65.2–92.3)
ECOG-Performans Status		
0	41	85.5
1	7	14.5
Tumor side		
Left	28	54.9
Right	23	45.1
Location		
Upper external quadrant	20	39.2
Upper internal quadrant	5	9.8
Lower internal quadrant	3	5.9
Lower external quadrant	3	5.9
Junction of external quadrant	6	11.8
Junction of internal quadrant	3	5.9
Junction of lower quadrant	3	5.9
Junction of upper quadrant	6	11.7
Periareolar	2	3.9
Median tumor size (mm)	12	(3–32)
Tumor stage		
T1a	28	54.9
T1b	18	35.3
T1c	5	9.8
Axillary lymph node status		
N0	46	90.1
N1mic	4	7.9
N1	1	2.0
Histology type		
Invasive ductal carcinoma	44	86.3
Invasive lobular carcinoma	3	5.9
Other	4	7.8
Histological grade		
1	32	62.7
2	14	27.4
3	5	9.8
Hormonal status		
Positive	48	94.1
Negative	3	5.9
Her-2 status		
Over-expressed	5	9.8
Non-over-expressed	46	90.2
Peri-neural invasion		
Yes	1	1.9
No	50	98.1
Median Ki-67 (%)	10	(5–60)
Median surg. Marg.(mm)	5	(1–10)
Implant time		
Intra operative	47	92.2
Post-operative	4	7.8
Median time interv. Surg./APBI (d)	7	(1–63)
Median number of vectors	11	(5–15)
Median number of planes	2	(1–3)
Median CTV (cc)	44	(11–124)
Median V100% (%)	96	(86–100)
Median V150% (%)	34	(23–48)
Median V200% (%)	12	(8–21)
Median DNR	0.35	(0.23–0.56)

Median time interv. Surg./APBI: median time between intervention and sfAPBI; Median surg. Marg.: median surgical margins; DNR: dose non-homogeneity ratio = V100/V150

### Treatment characteristics

A median number of 11 vectors [range: 5–15] on 2 planes [range: 1–3] were implanted (depending on the thickness and location of the target volume), mainly intra-operatively (92.2%). The median time between surgery and sfAPBI was 7 days [range: 1–63]. The median CTV was 44 cc [range: 11–124]. The median V100% was 96% [range: 86–100] (Table 1). The median treated volume was 42 cc [range: 10–124].

### Oncological outcome

**Fig. 1:**
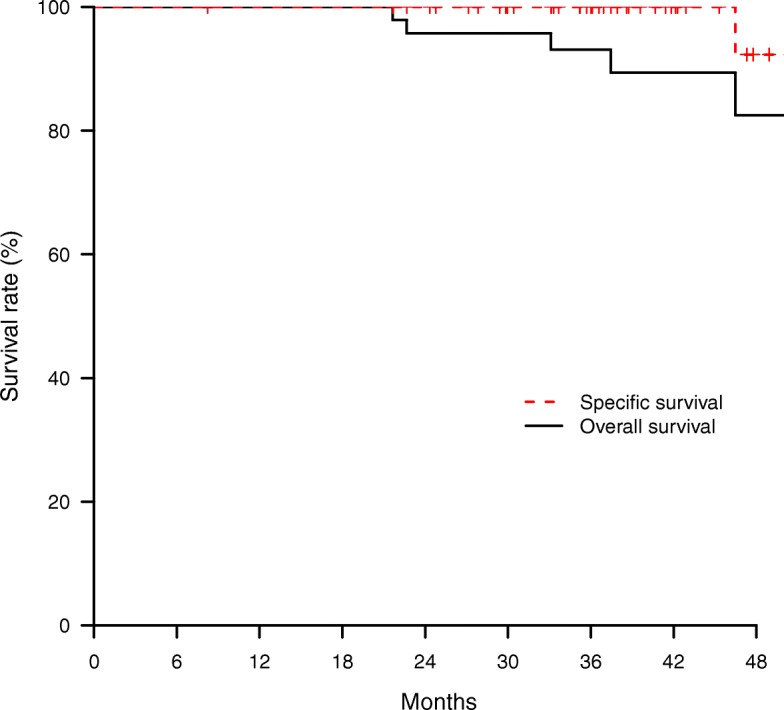
Specific and overall survival

With a median follow–up of 40 months [range: 36–42], no local relapse occurred while 1 pt. developed an axillary relapse (2.1%). Three-year LRFS, RRFS and SS were 100%, 3-year OS was 93.1% [86.4–1] (Fig. 1).

### Acute and late toxicity

Forty-five acute events were remained. The most frequent acute toxicity was grade (G) 1 hyperpigmentation (26.7%). Three pts. presented G3 acute toxicity (2 breast hematomas, 1 breast abscess). No ≥G3 late toxicity was observed while 15 late toxicities occurred (G1: 11 events [80%]). G1 breast fibrosis and hypopigmentation of puncture site were the most frequent late side effects. The rate of excellent cosmetic outcome was 76.4%. A breast asymmetry was noticed in 2 pts. (4%) (Table 2).

**Table 2:** Acute and late toxicity outcome

Toxicity	Number of events	%
Acute		
Grade 1		
Hyperpigmentation	12	26.7
Epithelitis	3	6.7
Breast hematoma	2	4.4
Other	13	28.9
Total	30	66.7
Grade 2		
Breast pain	2	4.4
Breast hematoma	2	4.4
Skin hyperpigmentation	2	4.4
Other	6	13.3
Total	12	26.7
Grade 3		
Breast hematoma	2	4.4
Breast infection	1	2.2
Total	3	6.7
Total number of events	45	100
Late		
Grade 1		
Breast fibrosis	4	26.7
Puncture site hypopig.	5	33.3
Telangectasia	2	13.3
Epithelitis	1	6.7
Other	1	6.7
Total	13	86.7
Grade 2		
Breast fibrosis	2	13.3
Total number of events	15	100
Cosmetic outcome		
Excellent	39	76.4
Good	12	25.6

Puncture site hypopig.: Puncture site hypopigmentation

## Discussion

APBI is now recognized as a validated irradiation option for low-risk breast cancer [12]. The current challenge is to deliver irradiation dose in the shortest treatment duration, in the most appropriate population. From a technical point of view, 2 different APBI approaches can be proposed: Intraoperative irradiation (electron or low-energy X photon [50 kV]) or postoperative irradiation either with brachytherapy (multicatheter interstitial brachytherapy [MIB] / balloon devices) or external beam irradiation (3D or Intensity modulated) [19]. Intraoperative irradiation (IORT) allows the optimal reduction of the treatment duration since the patient is irradiated during the lumpectomy process. However, at least 15% of patients are partially irradiated while the definitive histology is not suitable for this treatment [20]. Consequently, those patients need a post-operative whole breast irradiation (WBI) while the intra-op APBI is considered as a “boost”. Post-operative irradiation does permit treating only validated candidates for APBI due to an appropriate definitive pathological report compatible with APBI criteria while the number of transportation remains significantly higher compared to IORT. Furthermore, MIB-APBI allows best target coverage [21]. In this frame, a post-operative single fraction APBI appeared as an attractive technical option by drastically reducing the number of transportations and in the same time, alleviating the treatment related constraints mainly for elderly patients with frequent comorbidities [22].

In our study, after a median follow-up of 40 months, no local relapse was observed while SS rate was 100%. Two studies already reported oncological outcome after a MIB sfAPBI. In the SiFEBI prospective phase II trial, 26 elderly patients were treated with a 16 Gy sfAPBI. After a median follow-up of 37 months, there was no local relapse [10]. Recently, Latorre et al. reported the results of 20 pts. treated with a MIB sfAPBI of 18 Gy. After a median follow-up of 20 months, there was no local relapse [23]. Other teams investigated very hypofractionnated APBI, based on different regimens (1 to 7 fractions in 1 to 2 consecutive days) and HDR irradiation techniques (Per-operative and balloon devices) [24–27]. The results of those studies (oncological outcome and toxicities) are summarized in Table 3.

**Table 3:** Very hypo-fractionnated APBI discribed in the litterature

Authors	Year	# pts	MFU (months)	APBI techniques	Total dose (Gy)	D/f (Gy)	AG3 to (%)	LG3 tox (%)	LF (%)	RF (%)	DM (%)	Ex/good cosmetic result (%)
Sacchini	2008	18/34	31	HDR_IORT_	20/18	20/18	7.7	–	0	–	–	c
Khan	2013	30	11	Balloon^a^	28	7 (BID)	0	0	–	–	–	–
Wilkinson	2012/17	45	74	Balloon^b^	28	7 (BID)	13.3	2	0	0	0	91
Showalter	2016	28	6	HDR_IORT_	12.5	12.5	0	–	–	–	–	93
Hannoun-Levi	2017	26	37	HDR_MIB_	16	16	7.6	0	0	0	–	88
Latorre	2018	20	24	HDR_MIB_	18	18	0	0	0	0	5	80
Present study	2018	48	40	HDR_MIB_	16	16	6.3	0	0	0	0	100

^a^*Contura*™

^b^*Mammosite*™

^c^Cosmetic results were better with 18 Gy compared to 20 Gy

*# Pts*: number of patients; *MFU*: median follow-up; *APBI*: accelerated partial breast irradiation; *HDR*_*IORT*_: high-dose rate brachytherapy performed intraoperatively; *MIB*: multicatheter interstitial high-dose rate brachytherapy; *D/f*: dose per fraction; *AG3 tox*: acute ≥ Grade 3 toxicity; *LG3 tox:* late ≥ Grade 3 toxicity; *LF*: local failure; *RF*: regional failure; *DM*: distant metastasis; Ex/good cosmetic: percentages of excellent and good cosmetic results

Regarding MIB sfAPBI side effects, we did not report G ≥ 3 late toxicity confirming the safety of this approach already reported by others (Table 3). However, Sacchini et al. had to decrease the per-operative delivered dose from 20 Gy to 18 Gy due to the unexpectedly high-rate of acute toxicity.

Specifically for elderly patients presenting with a low-risk positive hormonal status breast cancer, the omission of adjuvant radiation therapy was suggested in order to alleviate the treatment. In those phase III randomized trials (Surgery + hormonal therapy with or without adjuvant WBI), there was no significant difference in terms of overall survival between irradiated and non-irradiated patients [28, 29]. However, there was a significant over-risk of local recurrence for patient without adjuvant breast irradiation. In elderly patients, the impact on functional status must be taken in account in treatment decision. The GERICO-O3 phase II trial aimed to evaluate the impact on functional status of MIB APBI in a cohort of 46 elderly women (median age: 74 years). Activity Daily Living (ADL) and Instrumental Activity Daily Living (IADL) scales were evaluated before and after ABPI. The scores remained unchanged at 6 and 12 months after APBI confirming no deleterious impact of MIB APBI [30].

## Conclusion

We reported promising and encouraging clinical outcome of a post-operative single fraction of MIB ABPI in the elderly. This approach leads to consider a sfAPBI as an attractive alternative to intra-operative radiation therapy while all the patients will be good candidates for APBI in regards to the post-operative pathological report. SfAPBI allows to drastically reducing the number of transportations and could benefit to the patients by decreasing fatigue and to the society by lowering cost related to transportations. More mature results (number of patients and follow-up) are needed to confirm the results.

## Abbreviations

ADL: Activity Daily Living
APBI: Accelerated partial breast irradiation
ASTRO: American Society of Radiation Oncology
DNR: Dose non-homogeneity ratio
GEC-ESTRO: Groupe Européen de Curiethérapie of the European Society for Radiotherapy and Oncology
HDR: High-dose rate
IADL: Instrumental Activity Daily Living
IORT: Intraoperative radiation therapy
LRFS: Local relapse-free survival
MIB: Multicatheter interstitial brachytherapy
OS: Overall survival
PS: Performans Status
RRFS: Regional relapse-free survival
sfAPBI: Single fraction APBI
SS: Specific survival
WBI: Whole breast irradiation
